# Prion Protein in Stem Cells: A Lipid Raft Component Involved in the Cellular Differentiation Process

**DOI:** 10.3390/ijms21114168

**Published:** 2020-06-11

**Authors:** Stefano Martellucci, Costantino Santacroce, Francesca Santilli, Valeria Manganelli, Maurizio Sorice, Vincenzo Mattei

**Affiliations:** 1 Department of Biotechnological and Applied Clinical Sciences, University of L’Aquila, 67100 L’Aquila, Italy; s.martellucci@sabinauniversitas.it; 2 Biomedicine and Advanced Technologies Rieti Center, “Sabina Universitas”, 02100, Rieti, Italy; costantinosantacroce@tiscali.it (C.S.); f.santilli@sabinauniversitas.it (F.S.); 3 Department of Experimental Medicine, “Sapienza” University, 00161 Rome, Italy; valeria.manganelli@uniroma1.it (V.M.); maurizio.sorice@uniroma1.it (M.S.)

**Keywords:** prion protein, prions, lipid raft, rafts, raft microdomains, stem cells, mesenchymal stem cells

## Abstract

The prion protein (PrP) is an enigmatic molecule with a pleiotropic effect on different cell types; it is localized stably in lipid raft microdomains and it is able to recruit downstream signal transduction pathways by its interaction with various biochemical partners. Since its discovery, this lipid raft component has been involved in several functions, although most of the publications focused on the pathological role of the protein. Recent studies report a key role of cellular prion protein (PrP^C^) in physiological processes, including cellular differentiation. Indeed, the PrP^C^, whose expression is modulated according to the cell differentiation degree, appears to be part of the multimolecular signaling pathways of the neuronal differentiation process. In this review, we aim to summarize the main findings that report the link between PrP^C^ and stem cells.

## 1. Introduction

The prion protein (PrP), a molecule discovered by Stanley Prusiner, is involved in the transmissible spongiform encephalopathies (TSEs), such as family fatal insomnia (FFI), Creutzfeldt–Jakob disease (CJD), Gerstmann–Sträussler–Scheinker (GSS), and other pathologies [1,2].

Many studies have shown that two possible protein tridimensional conformations may exist: the cellular prion protein (PrP^C^), normally expressed in all nucleated cells; and the scrapie prion protein (PrP^Sc^), which is involved in the TSEs. The main difference between these isoforms is the content of the β-sheets, which is higher in PrP^Sc^ compared to PrP^C^ [3,4,5]. 

In many conditions, the physiological isoform of PrP^C^ is able to translate into the pathological isoform PrP^Sc^; this transition can be regarded as a post-translational refolding process [6,7], leading to acquiring new physico-chemical properties. In fact, PrP^Sc^ has a high β-sheet structure, insolubility, and partial resistance against digestion by proteinase K [8,9,10].

In humans, PrP^C^ is encoded by the PRNP gene, and the protein consists of approximately 250 amino acid residues and a highly conserved membrane-bound glycoprotein anchored by glycosylphosphatidylinositol (GPI) [11].

In reference to the PrP^C^ structure, the N-terminus is distinguished by an octapeptide repeated region able to bind the Cu^2+^ ions and, for this reason, it is involved in oxidative stress resistance. The middle region contains a cluster of lysine residues and a hydrophobic domain [12], whereas the C-terminal globular domain contains 3 α-helices, 2 short β-sheets, and interconnecting loops [3,13,14]. Moreover, a disulfide bond is found between residues 179 and 214 [15] and, also, some N-linked glycans can be added at residues 181 and 197 [16].

In mammals, PrP^C^ is expressed in all nucleated cells although, it is mainly expressed in neuronal cells [17]. In response to some stimuli, such as proteases or reactive oxygen species (ROS) [18,19], the PrP^C^, like many other proteins, could be subjected to cleavage in different sites, giving rise to different membrane-bound and soluble fragments of different sizes and features [20].

Since its discovery, it has been well known that PrP^C^ is present on the plasma membrane associated with the lipid raft microdomains [21], structures that represent particular sub-compartments of the plasma membrane enriched in cholesterol and glycosphingolipids, such as GM3, GM1, and GD3 [22,23]. Several studies have shown that lipid rafts are important in the refolding process of the PrP^C^ in PrP^Sc^ [24,25]. Indeed, the increasing of the membrane-anchored PrP^C^ local concentration seems to be able to induce a conformational transition accompanied by di- or oligomerization of the PrP^C^. Elfrink et al. have proposed that membrane anchoring of an excess of prion protein is the structural prerequisite in the development of prion diseases [10,23]. Other authors have suggested that the prion protein–GM1 interaction within lipid rafts at the cell surface could play a significant role in the mechanism predisposing to pathology [26]. 

Although the PrP^Sc^ has been associated with the pathogenesis of TSEs, different reports provided evidences that PrP^C^ is a pivotal molecule with fundamental roles in various physiological and developmental processes [16,27]. 

Researchers focused on understanding the physiological role of the PrP^C^ [11]. In fact, since its discovery, many functions have been attributed to PrP^C^, such as the copper-binding ability in brain membrane fractions, which controls the activity of other membrane-associated copper-binding proteins and enhances copper incorporation into superoxide dismutase (SOD) [28,29]. 

Moreover, the preferential localization of PrP^C^ in the pre- and postsynaptic compartments of the nerve terminals implies that it might be involved in preserving the normal synaptic structure and function by regulating synaptic transmission and plasticity [30]. Recent evidence suggested the possibility that a hypoxia-mediated PrP^C^ increase plays an important role in angiogenesis [31,32]. 

Additionally, in vitro studies propose that PrP^C^ is involved both in the regulation of neuritogenesis [33,34] as well as axonal growth [35,36], and in tumorigenesis by regulating tumor growth, differentiation, and resistance to conventional therapies. In fact, PrP^C^ overexpression is related to the acquisition of a malignant phenotype of cancer stem cells (CSCs) in different tumors, such as pancreatic ductal adenocarcinoma (PDAC), osteosarcoma, breast cancer, gastric cancer, and glioblastoma multiforme (GBM) [37]. 

In the last years, several scientists have emphasized a possible role of PrP^C^ in stem cell biology [30,38]. Indeed, as reported in the literature, PrP^C^ is expressed in a wide variety of stem cells, including embryonic and hematopoietic stem cells, and its function has been linked to the modulation of the proliferation and self-renewal capacity of these kind of cells [38,39,40]. 

Tremblay et al. showed that PrP^C^ is firstly highly expressed during murine embryogenesis, predominantly in post-mitotic neural cells that have undergone neuronal differentiation [41]; subsequently, the PrP^C^ expression expands to non-neuronal tissues. In the human forebrain, PrP^C^ expression starts at the 11th week; it continues until the end of gestation and it occurs predominantly in the axonal tract, suggesting a specific role for this molecule in axonal growth during development [30,36].

A recent study published by Martellucci et al. has furnished evidence of the presence of PrP^C^ in human dental pulp-derived stem cells (hDPSCs) and of its role in the neuronal differentiation process; in addition, it has been shown that the lipid raft’s integrity is essential for the PrP^C^-induced signal pathways, and that is essential for the neuronal differentiation process of hDPSCs induced by the epidermal growth factor and basic fibroblast growth factor (EGF/bFGF) [42,43]. PrP^C^ plays a key role in the neuronal differentiation process [44], in which the PrP^C^ interacts with EGF-R within lipid rafts, playing a role in the multimolecular signal complexes involved in the hDPSCs neuronal differentiation process [42]. 

PrP^C^ is constitutively present in lipid rafts [45] and in a wide variety of stem cells [39,40]; in fact, it is involved in stemness modulation and, mostly, in the self-renewal and proliferation of tissue-resident stem cells and the neuronal differentiation of neural stem cells [46]. 

The purpose of this review is to highlight the role of PrP^C^ as a lipid raft component during neuronal and neuronal differentiation processes.

## 2. Prion Protein as Raft Component

Since its discovery, various authors have showed that PrP^C^ is present on the cell plasma membrane—associated with different gangliosides and cholesterol—within particular structures named “lipid raft microdomains” [21,47]. These structures represent domains of plasma membrane that contain high concentrations of cholesterol and glycosphingolipids, such as GM3, GM1, GD3, and proteins; lipid rafts can dissociate and associate rapidly, forming functional clusters in cell membranes [22,48]. They are distinct regions of the plasma membrane where they may represent a large fraction. Rafts are characterized by a distinctive protein and lipid composition, depending on the cell type or tissue [49].

A lot of proteins are present within lipid rafts; in particular, those involved in cell signaling. In fact, a number of proteins involved in the signal transduction pathways have been copurified with lipid rafts on a sucrose gradient as ZAP-70, Fyn, p56^lck^ [50,51]. For this reason, lipid rafts are thought to be involved in the regulation of signal transduction pathways [52,53].

These clusters, present in the outer monolayer of the plasma membrane, provide highly efficient lipid–protein modules, which operate in membrane trafficking and cell signaling [52]. Several authors theorized lipid rafts as sequestering platforms of specific proteins, thus modulating cell signaling [21,53,54].

Different evidence suggests that there are probably different mechanisms through which rafts may control cell signaling. For example, rafts may contain incomplete signaling pathways that are activated when a receptor is recruited within the raft or by suppressing the intrinsic activity of the signaling proteins present within rafts [55].

It was also demonstrated that some proteins are mainly distributed within lipid rafts or are recruited in response to specific stimuli [35].

Like other glycosylphosphatidylinositol (GPI)-anchored proteins, most PrP^C^ molecules are found in lipid rafts from neural and non-neural cells [56]. PrP^C^ is localized stably in the lipid raft microdomains and able to recruit the downstream signal transduction pathways by interaction with various partners [57]. 

**Figure 1:**
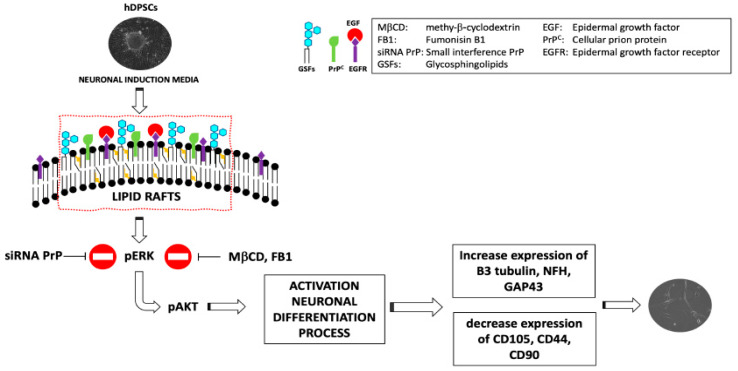
Role of lipid raft inhibitors and siRNA PrP in the neurodifferentiation process of hDPSCs.

In a recent study, it was reported that GM2 lipid rafts are present on the plasma membrane of hDPSCs. Besides, the PrP^C^ is able to associate specifically with GM2 in non-treated hDPSCs while, after the neuronal differentiation process induced by EGF/bFGF, the PrP^C^ associates preferentially with GD3 lipid rafts. Moreover, this study suggests that all the main lipid constitutive components of the rafts, such as the gangliosides and cholesterol, are essential for the hDPSCs neuronal differentiation process [35]. Thus, the results point out the functional role of the lipid rafts and PrP^C^ in the hDPSCs neuronal differentiation process, suggesting that these structures may represent specific chambers, where multimolecular signaling complexes, including the lipids (i.e., gangliosides, cholesterol) and proteins (i.e., PrP^C^, EGF-R), play a role in neuronal differentiation [22,35]. It was found that, following neuronal differentiation of the hDPSCs, EGF-R is recruited within the lipid raft where it interacts with PrP^C^ (Figure 1).

In fact, lipid raft inhibitors, such as fumonisin B1 and methyl-β-cyclodextrin (MβCD), significantly prevented ERK 1/2 and Akt phosphorylation and neuronal differentiation process induced by EGF/bFGF [35]. It was also observed that silencing of PrP^C^ by the usage of a specific small interference RNA (siRNA PrP), in order to ablate its function, affected neuronal differentiation process mediated by EGF/bFGF [35].

In agreement with this data, other authors highlighted the association of PrP^C^ with two components of the EGF-R macromolecular complex, such as Grb-2 [58] and Src [59]. This indicates that PrP^C^ may be part of the cell membrane complexes that regulate EGF/EGF-R signaling [42]. It was hypothesized that, after neuronal induction, EGF-R is recruited within the lipid rafts, where, interacting with PrP^C^ triggers the signal transduction, starting the neuronal differentiation process [42].

## 3. Prion Protein and Signaling Pathway 

PrP^C^ is a highly conserved cell surface GPI-anchored glycoprotein that, as reported above, was first identified as a molecule able to bind Cu^++^ [28]. Cross-linkage of GPI-anchored proteins usually results in protein sorting, shedding, and cell signaling, which is greatly influenced by the GPI-anchor signal sequence [60]. In particular, crosslink of PrP^C^ on the surface of T-lymphocytes has been associated with various cellular responses, such as the intracellular Ca^++^ mobilization [61], Src, and extracellular-signal-regulated kinase (ERK) activation or capping of the lipid raft microdomains with Fyn phosphorylation [62]. Indeed, anti-PrP^C^ antibody coimmunoprecipitated Fyn; as a downstream target of Fyn activation, PrP^C^ was shown to activate the ERK1/2, which takes part in the mitogen-activated protein kinase (MAPK) cascades [63]. Recently, Martellucci et al. confirmed and extended the role of PrP^C^ in this signaling pathway, demonstrating that recombinant prion protein 23–231 (recPrP^C^) is involved in the neuronal differentiation process, by activating ERK 1/2 and Akt [35,42]. Interestingly, this activity required an endogenous PrP^C^ to mediate the way of the signal triggered by recPrP^C^. Furthermore, PrP^C^ was shown to activate the Lyn- and Syk-dependent signal transduction pathways in different cell types [51,64]. It leads to a transient release of Ca^++^ that induces activation of protein kinase C- and Ca^++^-dependent tyrosine kinases. In addition, a direct interaction of PrP^C^ with synapsin Ib and the adapter protein Grb-2 was also reported [58]. 

PrP^C^ may also activate the neuroprotective signaling pathway(s). In fact, dimerization of PrP^C^ leads to clustering in multimolecular complexes and serves to regulate different aspects of neuronal homeostasis, whereas intracellular dimerization appears to be the most relevant event in neuroprotection, via N1 and C1 prion metabolites [23]. Indeed, the dimerization stimulates α-cleavage and thus the production of the neuroprotective fragments.

The neuroprotective functions of PrP^C^ may be attributed to its BCL-2-like properties [65]. PrP^C^ protects against cell death by preventing the conformational change of BAX occurring during BAX activation [66]. A neuroprotective activity of PrP^C^ was reported by Mitteregger et al. [67], who revealed that both the C-terminal GPI anchor and the N-terminal domain are required for this physiological activity. In particular, the cAMP-dependent protein kinase A (PKA) seems to mediate the neuroprotective signals, as demonstrated by Chiarini et al. [68]. 

In addition, the phosphatidylinositol 3-kinase/AKT (PI3K/AKT) pathway is involved in the regulation of PrP^C^-induced neuroprotection. Thus, PrP^C^ might act as a signaling molecule at the cell surface to promote stress-protective signaling under physiological conditions, which can be switched to toxic signaling through the interaction with β-sheet-rich conformers. In this concern, the role of PrP^C^ may depend on its intracellular localization [61]. Indeed, PrP^C^ translocation from lipid rafts to non-lipid rafts prevents p38 and caspase-3 activation with consequent inhibition of cell apoptosis. PrP^C^ is generally reported as a plasma membrane protein; however, studies revealed the presence of endogenous PrP^C^ as an interacting protein with the membrane/organelles [69], as well as with the cytoskeleton network. In fact, lipid microdomains are similarly formed at subcellular organelles, including the endoplasmic reticulum, Golgi complex, and mitochondria, named lipid raft-like microdomains [70]. In the last few years, Mattei et al. identified PrP^C^ as a new component of mitochondrial raft-like microdomains in T cells undergoing CD95/Fas-mediated apoptosis, indicating that PrP^C^ could undergo intracellular re-localization via the ER–mitochondria-associated membranes (MAM) and microtubular network [69].

A new and innovative point of view suggests that PrP^C^ increases the calcineurin activity, resulting in decreased AMPK phosphorylation that induces autophagic cell death [71]. Interestingly, this study demonstrated that the prion protein–calcineurin activation was involved not only in prion protein-mediated neuronal cell death but also in the AMPK and autophagy signaling pathways. It regulates metabolic homeostasis [72] by controlling autophagy [73]. However, the overexpression of PrP^C^ inhibited the autophagic flux signals, lipid accumulation, as well as the PPAR-γ and C/EBP-α mRNA and protein expression levels in comparison to the control cells [74]. 

In conclusion, the modular structure, the variety of binding partners, and the typical localization within the lipid rafts, suggest that PrP^C^ may be a key component of the dynamic platforms on the cell surface, with the capability to assemble multicomponent complexes through different domains, triggering different signaling pathways that regulate differentiation and cell fate. 

## 4. Prion Protein and Stem Cells

Different studies demonstrated a strong relationship between the cellular form of prion protein and stem cells, considering the PrP^C^ as an element of the pluripotency and self-renewal matrix [38,75]. Furthermore, the expression of PrP^C^ is modulated according to the degree of stem cell differentiation and it is involved in the molecular signaling that underlies the differentiation process of several cell lineages [42,76].

In human embryonic stem cell (hESC) differentiation, it was observed that PrP^C^ is involved in controlling the cell cycle dynamics, self-renewal, and the fate of this kind of cell. Furthermore, silencing PrP^C^ in hESCs undergoing spontaneous differentiation altered the dynamics of the cell cycle and changed the balance between the lineages of the three germ layers, where differentiation toward ectodermal lineages was suppressed. Moreover, over-expression of PrP^C^ in hESCs undergoing spontaneous differentiation inhibited differentiation toward lineages of all three germ layers and helped to preserve high proliferation activity. These results illustrate that PrP^C^ is involved in key activities that dictate the status of the hESCs, including regulation of the cell cycle dynamics, controlling the switch between self-renewal and differentiation, and determining the fate of hESCs differentiation. Thus, PrP^C^ is at the crossroads of several signaling pathways that regulate the switch between preservation of or departure from the self-renewal state, control cell proliferation activity, and define the stem cell fate [39]. 

In human mesenchymal stem cells (hMSCs), the PrP^C^ has been shown to enhance proliferation and promote self-renewal of this kind of cell. In fact, the expression of PrP^C^ decreased in hMSCs following ex vivo expansion. When PrP^C^ expression was knocked down, the hMSCs showed a significant reduction in proliferation and differentiation. In contrast, the hMSCs expanded in the presence of small molecule 3/689, a modulator of PrP^C^ expression, showing retention of PrP^C^ expression with ex vivo expansion and an extended lifespan of up to 10 population doublings [76]. On the basis of this findings, a lot of preclinical and clinical studies indicated PrP^C^ as a potential target for therapeutic strategy.

Indeed, as reported by Lee et al.*,* hMSCs are promising candidates for stem cell-based therapy in ischemic diseases that induce pathophysiological conditions, such as oxidative stress and inflammation. The authors demonstrated how melatonin promotes hMSCs functionality and enhances MSC-mediated neovascularization in ischemic tissues through the upregulation of PrP^C^ expression. So, melatonin-treated hMSCs could provide a therapeutic strategy for vessel regeneration in ischemic disease, and the targeting of PrP^C^ levels may prove instrumental for MSC-based therapies [77]. In reference to melatonin, another work team showed that this hormone inhibits colon cancer stem cells (CSCs) by regulating the PrP^C^–Oct4 axis. Indeed, in specimens from patients with colorectal cancer, the expressions of PrP^C^ and Oct4 were significantly correlated with metastasis and tumor stages. Co-treatment with 5-fluorouracil (5-FU) and melatonin inhibited the stem cell markers Oct4, Nanog, Sox2, and ALDH1A1 by downregulating PrP^C^. In this way, tumor growth, proliferation, and tumor-mediated angiogenesis were suppressed. In colorectal CSCs, PRNP overexpression protects Oct4 against inhibition by 5-FU and melatonin. So, the authors suggest that the co-treatment with anticancer drugs and melatonin is a potential therapy for colorectal cancer and PrP^C^ maintains cancer stemness during tumor progression. Therefore, targeting the PrP^C^–Oct4 axis may prove instrumental in colorectal cancer therapy [78]. 

In the same direction of Lee et al., many studies demonstrated that MSCs promote regeneration of injured tissues, interacting with the PrP^C^ that plays an active role in neuronal survival and angioneurogenesis [77,78,79,80]. In fact, hypoxia enhanced the proliferative potential of MSCs by promoting the expression of normal PrP^C^, suggesting that hypo-MSCs offer a therapeutic strategy for accelerated neovasculogenesis in ischemic diseases, and that PrP^C^ comprises a potential target for MSC-based therapies [81]. Corsaro et al. also showed that PrP^C^ regulates different biological functions in human tumors, including glioblastoma (GBM). The authors analyzed the role of PrP^C^ in GBM cell pathogenicity, focusing on tumor-initiating cells (TICs or CSCs), the subpopulation responsible for development, progression, and recurrence of most malignancies. Analyzing four GBM CSC-enriched cultures, they showed that PrP^C^ expression is directly correlated with the proliferation rate of the cells. To better define its role in CSCs biology, they knocked-down PrP^C^ expression in two of these GBM-derived CSCs cultures by specific lentiviral-delivered shRNAs. The work provided evidence that the CSC proliferation rate, spherogenesis, and in vivo tumorigenicity are significantly inhibited in PrP^C^ downregulated cells. Moreover, PrP^C^ downregulation caused loss of expression of the stemness and self-renewal markers (NANOG, Sox2) as well as the activation of differentiation pathways (i.e. increased GFAP expression). The authors suggested that PrP^C^ controls the stemness properties of human GBM CSCs and that its downregulation induces the acquisition of a more differentiated and less oncogenic phenotype [82].

## 5. Prion Protein in Neural and Neuronal Differentiation Processes

The spectrum of proposed biological functions of PrP^C^ has been expanded rapidly over the last decade. Extensive experimental works disclosed multiple physiological roles of PrP^C^ at the molecular, cellular, and systemic levels, affecting the homeostasis of copper, neuroprotection, stem cell renewal, and memory mechanisms, among others. Various authors proposed that the biological function of the PrP^C^ is that of a cell surface scaffold protein, based on the striking similarities of its functional properties with those of scaffold proteins involved in the organization of intracellular signal transduction pathways [57,83]. However, PrP^C^ is highly conserved in mammals and is present on all nucleated cells, although it is mainly expressed in the central and peripheral nervous system. So, an increasing number of authors investigated the role of PrP^C^ as a key component of multimolecular complexes during the neuronal differentiation process [43]. As reported by Lee et al., PrP^C^ is a glycoprotein that is expressed on the cell surface beginning with the early stages of embryonic stem cell differentiation. The ectopic expression of PrP^C^ in ESCs triggers differentiation toward endodermal, mesodermal, and ectodermal lineages, whereas silencing of PrP^C^ suppresses the differentiation toward ectodermal but not endodermal or mesodermal lineages [39]. Starting with the role of PrP^C^ in controlling the balance between cells of different lineages, the authors also tested whether PrP^C^ controls the differentiation of hESCs into cells of the neuron-, oligodendrocyte-, and astrocyte-committed lineages. They found that silencing of PrP^C^ suppressed the differentiation toward all three lineages. Moreover, switching PrP^C^ expression during a differentiation time course revealed that silencing PrP^C^ expression during the very initial stage that corresponds to embryonic bodies has a more significant impact than silencing it at the later stages of differentiation. Their work illustrated that PrP^C^ controls differentiation of hESCs toward the neuron-, oligodendrocyte-, and astrocyte-committed lineages, and is likely involved at the stage of uncommitted neural progenitor cells rather than lineage-committed neural progenitors [46]. The importance of PrP^C^ at the early stages of neural differentiation is represented by different studies. 

Prodromidou et al. showed that PrP^C^ is essential for proper neural stem/precursor cells (NPCs) proliferation, neuronal differentiation, and, moreover, PrP^C^ is required for the NPC response to the neural cell adhesion molecule. In the absence of PrP^C^, NCAM not only fails to promote neuronal differentiation but also induces an accumulation of doublecortin-positive neuronal progenitors at the proliferation stage. So, the authors demonstrated that PrP^C^ plays a critical role in neuronal differentiation of the NPCs and suggest that this function is, at least in part, NCAM-dependent [84]. Steele et al. investigated the role of PrP^C^ in neural development in adult neurogenesis, which occurs constitutively in the dentate gyrus of the hippocampus and in the olfactory bulb from precursors in the subventricular zone rostral migratory stream. Loss and gain-of-function experiments demonstrate that the PrP^C^ levels correlate with the differentiation of multipotent neural precursors into mature neurons in vitro, and that the PrP^C^ levels positively influence neuronal differentiation in a dose-dependent manner [85]. 

Taken together, this data suggests that PrP^C^ plays a common and important role in the commission of NPCs toward the neuron-, oligodendrocyte-, and astrocyte lineages; indeed, silencing of the PRNP gene prevents both the neural and neuronal differentiation process.

Recent studies show that both the recombinant form [35] and cleavage products of PrP^C^ [86] are involved in the neuronal differentiation process and neuritogenesis. Martellucci et al. demonstrated that recPrP^C^ was able to activate the neuronal differentiation process and induce the expression of the typical neuronal markers, such as β3-Tubulin, NFH, and GAP-43; in fact, the authors reported that, when the PrP^C^ was silenced by siRNA, the neuro-induction was blocked. Furthermore, lipid raft inhibitors, such as fumonisin B1 and MβCD, significantly prevented the neuronal differentiation process, suggesting that lipid raft integrity plays a key role in recPrP activity [34]

Furthermore, Collins et al. identified a switch between neural stem cell (NSC) proliferation and quiescence through the changing intracellular redox signaling, showing that N-terminal post-translational cleavage products of the PrP^C^ induce a quiescent state, halting the NSCs’ cellular growth, migration, and neurite outgrowth. Quiescence is initiated by the PrP cleavage products through reducing the intracellular levels of reactive oxygen species. First, inhibition of redox signaling results in increased mitochondrial fission, which rapidly signals quiescence. Thereafter, quiescence is maintained through downstream increases in the expression and activity of superoxide dismutase-2, which reduces mitochondrial superoxide. Besides, the authors observed that PrP is predominantly cleaved in quiescent NSCs, indicating a homeostatic role for this cascade [86]. 

Thus, the shed PrP^C^ and its cleavage products are biologically active fragments that may potentially participate with other biological processes, such as the differentiation process. In fact, proteolytic cleavage events may alter either the biological functions of PrP^C^ or produce protein fragments harboring specific intrinsic properties, thus contributing to a higher biological complexity.

## 6. Conclusions Remarks

The prion protein is an enigmatic protein with a pleiotropic effect on different cell types. It was previously shown to play a key role in some physiological processes, including cellular activation, apoptosis, and differentiation, by functional interaction with a multimolecular signaling complex through lipid rafts. Recent evidence pointed out its role in stem cell differentiation, where it appears to be involved in the molecular signaling of neuronal differentiation.
